# Microbial Gene Profiling and Targeted Metabolomics in Fecal Samples of Dogs With Chronic Enteropathy With or Without Increased Dysbiosis Index

**DOI:** 10.1111/jvim.70199

**Published:** 2025-08-14

**Authors:** Chih‐Chun Chen, Rachel Pilla, Linda Toresson, Chi‐Hsuan Sung, Amanda B. Blake, Bruna Correa Lopes, Jonathan Turck, Albert E. Jergens, Stacie C. Summers, Stefan Unterer, Patricia Eri Ishii, Paula R. Giaretta, M. Katherine Tolbert, Jan S. Suchodolski

**Affiliations:** ^1^ Gastrointestinal Laboratory, School of Veterinary Medicine and Biomedical Sciences Texas A&M University College Station Texas USA; ^2^ Evidensia Specialist Animal Hospital Helsingborg Sweden; ^3^ Department of Veterinary Clinical Sciences, College of Veterinary Medicine Iowa State University Ames Iowa USA; ^4^ Carlson College of Veterinary Medicine, Oregon State University Corvallis Oregon USA; ^5^ Clinic for Small Animal Internal Medicine, Vetsuisse Faculty University of Zurich Zurich Switzerland; ^6^ Stonewell Gastrointestinal Vet São Paulo Brazil

**Keywords:** bile acids, carbohydrate metabolism, DNA shotgun sequencing, gas chromatography–mass spectrometry, lipid metabolism, malabsorption

## Abstract

**Background:**

In previous studies, only a subset of dogs with chronic enteropathy (CE) had an increased dysbiosis index (DI) or altered fecal metabolites or both, suggesting differences in underlying intestinal pathophysiology between these subsets.

**Objectives:**

To compare microbial functional genes and fecal metabolites between healthy dogs with DI < 0 (HC) and dogs with CE and DI > 0 (increased DI‐CE) or DI < 0 (normal DI‐CE).

**Animals:**

Retrospective cross‐sectional study including 78 HC and 138 ce dogs.

**Methods:**

Fecal microbiome was assessed by DNA shotgun sequencing. Dysbiosis index was quantified by qPCR. Targeted fecal metabolites, long‐chain fatty acids, sterols, bile acids (BAs), and carbohydrates were measured using gas chromatography–mass spectrometry (GC–MS).

**Results:**

In permutational analysis of variance (PERMANOVA), functional gene profiles showed larger shifts in increased DI‐CE (median *R* ^2^ [95% confidence interval (CI)] = 0.12 [0.08–0.17]) than normal DI‐CE (0.02 [0.01–0.04]) compared with HC (adjusted‐*p* < 0.02), characterized by increased counts of carbohydrate and lipid degradation genes. Similarly, increased DI‐CE (PERMANOVA, median *R* ^2^ [95% CI] = 0.23 [0.14–0.34]) had larger shifts in fecal metabolome than normal DI‐CE (0.10 [0.04–0.20]; adjusted‐*p* < 0.02). Increased DI‐CE had lower fecal unconjugated secondary BAs percentage (95% CI; HC, 88.4%–96.4%; normal DI‐CE, 79.8%–99.0%; increased DI‐CE, 28.1%–64.1%) and transporter‐independent carbohydrates (combined ribose, xylose, rhamnose, and arabinose) concentrations (1.6–2.6; 0.7–1.8; 0.3–1.3 ng/mg; adjusted‐*p* < 0.01).

**Conclusions:**

Results indicate differences in fecal microbial gene profiles and metabolome in increased DI‐CE versus normal DI‐CE and HC, suggesting dogs with an increased DI have more severe intestinal changes in metabolic functions.

**Abbreviations**
BA: bile acid
CE: chronic enteropathy
DI: dysbiosis index
GC–MS: gas chromatography–mass spectrometry
HC: healthy control dogs
IBD: inflammatory bowel disease
Increased DI‐CE: dogs with chronic enteropathy with abnormal dysbiosis index (DI > 0)
Normal DI‐CE: dogs with chronic enteropathy with normal dysbiosis index (DI < 0)

## 1 Introduction

Intestinal dysbiosis can be broadly defined as alterations in microbial composition and function. In humans with inflammatory bowel disease (IBD), typical changes include a decrease in microbial diversity, a decrease in short‐chain fatty acid‐producing bacteria such as *Faecalibacterium*, an increase in pathobionts such as *Escherichia coli* , and functional changes such as decreased conversion from primary to secondary bile acids (BAs) [1]. Very similar changes also have been reported in dogs with chronic enteropathy (CE) [2, 3, 4, 5, 6, 7, 8]. A recent meta‐analysis reported that, similar to humans, decreased microbial diversity, an increased dysbiosis index (DI) [9], including decreased *Faecalibacterium* and increased *E. coli* , and a decreased secondary BAs percentage are associated with CE in dogs [10].

Although the fecal microbiome in dogs can be impacted by various factors such as age [11], diet [12, 13, 14], and microbiome modulators [15, 16], these changes are typically smaller compared with those associated with intestinal disease or antibiotic use [17]. Therefore, in those dogs with severe shifts in the microbial composition compared to healthy dogs, intestinal structural damage, dysmetabolism, and inflammation are suspected [17]. Multiple studies have found that only a subset of dogs with CE has significant alterations in fecal microbial and metabolomic profiles, suggesting different underlying intestinal changes and disease subtypes in dogs with CE [2, 4, 8, 18, 19, 20, 21]. Cobalamin deficiency has been reported in a subset of dogs with CE [22], and was associated with more severe microbial shifts [23]. Additionally, initial studies indicate that dogs with increased DI had a short‐lasting response to fecal microbiota transplantation and recurrence of dysbiosis compared with dogs with no or mild changes in the DI [24, 25, 26, 27]. Furthermore, increased DI is associated with more severe shifts in the intestinal microbiota based on next‐generation sequencing [17, 28]. These findings suggest that DI could serve as a functional tool for subcategorizing dogs with CE and potentially identifying groups with different pathophysiology.

Although most carbohydrates, fats, and proteins are digested and absorbed by the host, a small fraction, primarily dietary fiber, reaches the colon for microbial fermentation. This proportion may increase in malabsorptive or maldigestive conditions. Alterations in host and microbial‐derived metabolites (e.g., BAs, long‐chain fatty acids, sterols, monosaccharides) have been described in CE in targeted or untargeted metabolomics studies [2, 3, 4, 5, 6, 7, 8, 18, 20, 29]. Alterations in fecal metabolomic profiles may occur before dogs present with clinical signs [30]. A previous study showed lower abundances of predicted microbial amino acid pathways in dogs with IBD [4]. One study of host gene expression on small intestinal biopsy samples reported fatty acid uptake and transport genes being underexpressed in dogs with CE [31]. This finding emphasizes that alterations in dogs with CE occur in both the host and microbiome. Identifying different patterns in host and microbial functions will allow a better understanding of subsets of intestinal dysfunction and may allow individualized interventions for dogs with CE.

We aimed to explore the following objectives in dogs with CE and normal versus increased DI: (1) the microbial gene composition by analyzing the functional gene profile and pathways, (2) the taxonomy profiles by analyzing the microbial composition from DNA shotgun sequencing and qPCR assays, and (3) selected fecal metabolites utilizing targeted gas chromatography–mass spectrometry (GC–MS) assays.

## 2 Materials and Methods

### 2.1 Study Population

Fecal samples were retrospectively selected from previous studies collected at different institutions from 2014 to 2022 (Table S1) [17]. Our study included fecal samples from 189 dogs, comprising 74 clinically healthy control (HC) dogs with a normal DI and 115 dogs with CE. Dogs in the HC group were evaluated as clinically healthy by veterinarians based on physical examinations with body condition scores (BCSs) obtained using World Small Animal Veterinary Association (WSAVA) guidelines. These HC dogs had not received any antibiotics, antacids, anti‐inflammatory medications, or corticosteroids within the past 6 months. Among the HC dogs, canine inflammatory bowel disease activity index (CIBDAI) [32] was assessed in 23 dogs. The diagnostic evaluation of dogs with CE followed standardized protocols as described previously [24]. In brief, dogs were included if they presented with gastrointestinal signs (e.g., vomiting, diarrhea, hyporexia, anorexia, weight loss) for at least 3 weeks and extragastrointestinal causes had been ruled out. Those with antibiotic exposure in the past 4 weeks were excluded. Among the CE group, CIBDAI was documented in 92 dogs.

Dogs with CE were further categorized into groups based on their DI. First, 57 dogs with CE and a DI < 0 were classified as normal DI‐CE, and the other 58 dogs with CE and a DI > 0 were classified as increased DI‐CE.

Among the 115 dogs with CE, 85 had clinical phenotypes assigned by the clinician: 22 dogs were recorded as having food‐responsive enteropathy, 34 as having steroid‐responsive enteropathy, 20 as having non‐responsive enteropathy, and 6 as having protein‐losing enteropathy. The other 33 dogs lacked specific clinical phenotypes. At least two diet trials were conducted before ruling out food responsiveness.

Feces were collected within 24 h after defecation and immediately stored at either −20°C or −80°C. Feces then were transported on dry ice to a central laboratory (Gastrointestinal Laboratory at Texas A&M University), where samples were subsequently stored at −80°C until further analysis.

### 2.2 Fecal Dysbiosis Index

Fecal DNA extraction was performed using the MoBio Power Soil DNA Isolation Kit (MO BIO Laboratories Inc., Carlsbad, CA, USA), following the manufacturer's instructions. The qPCR assays and DI calculation algorithm were described previously [9]. This assay quantified total bacteria, *Blautia, Peptacetobacter (Clostridium) hiranonis, E. coli, Faecalibacterium, Fusobacterium, Streptococcus*, and *Turicibacter*. Additionally, *Bifidobacterium* and *Bacteroides* also were quantified by qPCR assays as described previously [17].

### 2.3 Shotgun Metagenomic Sequencing

Shotgun metagenomic DNA sequencing was performed at Diversigen (New Brighton, MN, USA). A procedure adapted from the Nextera XT kit (Illumina, San Diego, CA, USA) was used to prepare libraries. With an aimed mean target depth of 2 million reads per sample, libraries were sequenced on an Illumina NovaSeq 6000 using paired‐end 2 × 150 reads. Both positive and negative controls were included on each DNA extraction and library preparation plate. Host reads were removed against a canine database using bowtie2. The DNA sequences were processed through the HUMAnN3 pipeline (version 3.8) under default settings [33]. For taxonomic profiling, sequences were mapped against the MetaPhlAn3 gene database (mpa_v30_CHOCOPhlAn_201901) to generate relative abundance and estimated count outputs. For functional gene profiles, the UniRef90 database was used to provide gene families definitions. Then, the gene families were mapped to pathways definitions from the MetaCyc database (version 24.0) incorporated in the HUMAnN3 pipeline. This pipeline also stratified gene families and pathways to determine the contribution of individual microbe species.

### 2.4 Fecal Targeted Metabolomics

#### 2.4.1 Fatty Acids, Sterols, and Bile Acids

Fecal concentrations of fatty acids, sterols, and BAs were measured in a subset of fecal samples: 22 HC dogs, 27 normal DI‐CE, and 20 increased DI‐CE. A previously documented GC–MS quantitative assay [20, 34] was performed to assess these metabolites: long‐chain fatty acids (i.e., palmitic acid, linoleic acid, α‐linolenic acid, oleic acid, cis‐vaccenic acid, stearic acid, arachidonic acid, gondoic acid, erucic acid, docosanoic acid, and nervonic acid), zoosterols (i.e., cholesterol, coprostanol, cholestanol, and lathosterol), phytosterols (i.e., β‐sitosterol, brassicasterol, campesterol, fucosterol, sitostanol, and stigmasterol), and unconjugated BAs (i.e., cholic acid, chenodeoxycholic acid, lithocholic acid, deoxycholic acid, and ursodeoxycholic acid).

#### 2.4.2 Carbohydrates

The fecal concentrations of glucose, galactose, and mannose, fructose, ribose, rhamnose, xylose, and arabinose were assessed in 92 dogs with sufficient fecal samples: 29 HC dogs, 27 normal DI‐CE, and 36 increased DI‐CE. A GC–MS protocol described previously for sugar quantitation in plant leaves was adapted and modified [35]. An aliquot of 10 mg lyophilized sample was weighed in a 2 mL tube with weight recorded to calculate concentrations in μg/mg of lyophilized feces. In each sample, 500 μL dimethyl sulfoxide was added and agitated for 15 min at 65°C at 1000 relative centrifugal force (rcf) using an Eppendorf thermomixer C (Eppendorf, Hamburg, Germany). A mixture of 20 μL 1,6‐anhydro‐beta‐d‐glucose (200 μg/mL) and 480 μL dimethyl sulfoxide was added to each sample, followed by shaking three times for 30 s each time using a Fisherbrand bead mill 24 homogenizer (Fisher Scientific, Hanover Park, Illinois). The homogenate was agitated for 1 h at 40°C with an Eppendorf thermomixer C and then centrifuged at 10 000 rpm for 3 min. Two‐hundred microliters of the supernatant were transferred to a 7 mL glass tube. Then, 30 μL of 1‐methylimidazole and 150 μL of acetic anhydride were mixed with the supernatant and incubated at room temperature for 30 min. Six‐hundred microliters of Milli‐Q water then were added to remove the unreacted acetic anhydride, and the acetyl derivatives were extracted with 400 μL of ethyl acetate. Finally, 1 μL of the upper organic extract was injected into the GC–MS for analysis. All of the chemical compounds and reagents were purchased from a commercial supplier (Sigma‐Aldrich, St. Louis, Missouri).

A GC–MS (6890 N and 5975 inert Mass Selective Detector; Agilent) equipped with an autosampler (7683 Series; Agilent) was used. A capillary column (DB‐1 ms Ultra Inert; Agilent) was used with the following dimensions: length: 30 m, diameter: 0.250 mm, film: 0.25 μm. A 100:1 split ratio was utilized after a 1 μL sample injection with an inlet temperature of 250°C. After injection, the oven temperature started at 50°C, ramped at 12°C/min to 180°C, and ramped to 190°C at 5°C/min. After holding at 190°C for 6 min, the temperature ramped at 30°C/min to 250°C. After data acquisition, the oven was heated to 325°C for 3 min for postrun column cleaning. Helium was used as the carrier gas at a nominal flow rate of 1 mL/min. Mass spectral data was analyzed using Agilent MassHunter Quantitative Analysis (MS).

The assay was analytically validated for accuracy, precision, and reproducibility. Calibration curve recovery and spiking recovery were calculated as (observed value [μg/mL]/expected value [μg/mL]) × 100%. To evaluate precision and reproducibility, inter‐assay and intra‐assay variabilities were assessed by calculating the coefficients of variation (CV% = [SD/mean] × 100%). Validation results and more details can be found in Table S2. Upper and lower limits of quantification were established by a standard curve development that spanned the working range of the assay useful in detecting a variety of glucose concentrations from over 50 dogs from unrelated studies.

### 2.5 Statistical Analyses

The Bray–Curtis dissimilarity matrix was used to calculate the beta diversity of the microbial compositions, pathway profiles, and targeted microbial and metabolomic profiles, which then were visualized using principal coordinates analysis (PCoA). Subsequently, permutational multivariate analysis (PERMANOVA) [36] was employed to compare the differences in beta diversity between groups. Then, the influence of targeted microbial and metabolomic variables on the ordination was tested using the envfit function in R, with the squared correlation coefficient (*R* ^2^) calculated to quantify the proportion of variance in the ordination explained by each variable. Microbiome multivariable associations with linear models (MaAsLin2) [37] were used to discover differential pathways between groups. Statistical significance and coefficient values representing the magnitude and direction of the association between features were calculated by MaAsLin2. GraphPad Prism (version 10.2.0) was employed to generate graphs showing the relative abundance of these pathways in each group, with adjusted‐*p* values derived from MaAsLin2. All of the analyses described above and part of the visualizations were performed in R software (version 4.4.0) with the following packages: vegan (version 2.6–4) [38], Maaslin2 (version 1.18.0), and ggplot2 (version 3.5.1). The Kruskal–Wallis test followed by Dunn's test was used to compare fecal concentrations of metabolites between groups. Spearman's correlation was used to assess the correlation between concentrations of fecal metabolites and DI. Fisher's exact test was used to compare sex distribution between HC and CE groups and between the two CE subgroups. Kruskal–Wallis tests, Dunn's tests, Spearman's correlations, Fisher's exact test, and their visualizations were performed using GraphPad Prism. Results were considered significant when adjusted‐*p* value < 0.05 in PERMANOVA, MaAsLin2, Dunn's test, Fisher's exact test, and Spearman's correlations.

## 3 Results

### 3.1 Study Population

Among 173 dogs with available sex information, male dogs were more common in the CE group (36 female and 65 male dogs; 36% and 64%, respectively) compared with the HC group (41 female and 31 male dogs; 57% and 43%, respectively; *p* = 0.01). Dogs with CE were significantly older than HC dogs (*p* < 0.01), with a median age of 6 years (range, 0.8–15) and 2.7 years (range, 0.5–13), respectively. However, no significant sex (adjusted‐*p* = 0.22) and age (adjusted‐*p* = 0.18) differences were found between normal DI‐CE and increased DI‐CE. The HC dogs had significantly lower body weight (median [range]: 9.9 [3.2–39.2] kg) compared to dogs with CE (19.7 [2.7–65.8] kg; *p* < 0.01). However, no significant difference (*p* = 0.12) was found in BCS between HC dogs (median [range]: 5 [4–7]) and dogs with CE (5 [3–7]). Our study population comprised dogs of various breeds living in diverse geographical locations and fed various diets. The CIBDAI score was significantly higher in dogs with CE (6 [0–15]) compared with the HC group (1 [0–2]; *p* < 0.01). However, no difference was found between normal DI‐CE (6 [0–13]) and increased DI‐CE (6 [0–15]; *p* > 0.99).

### 3.2 Taxonomy Profiles

**FIGURE 1:**
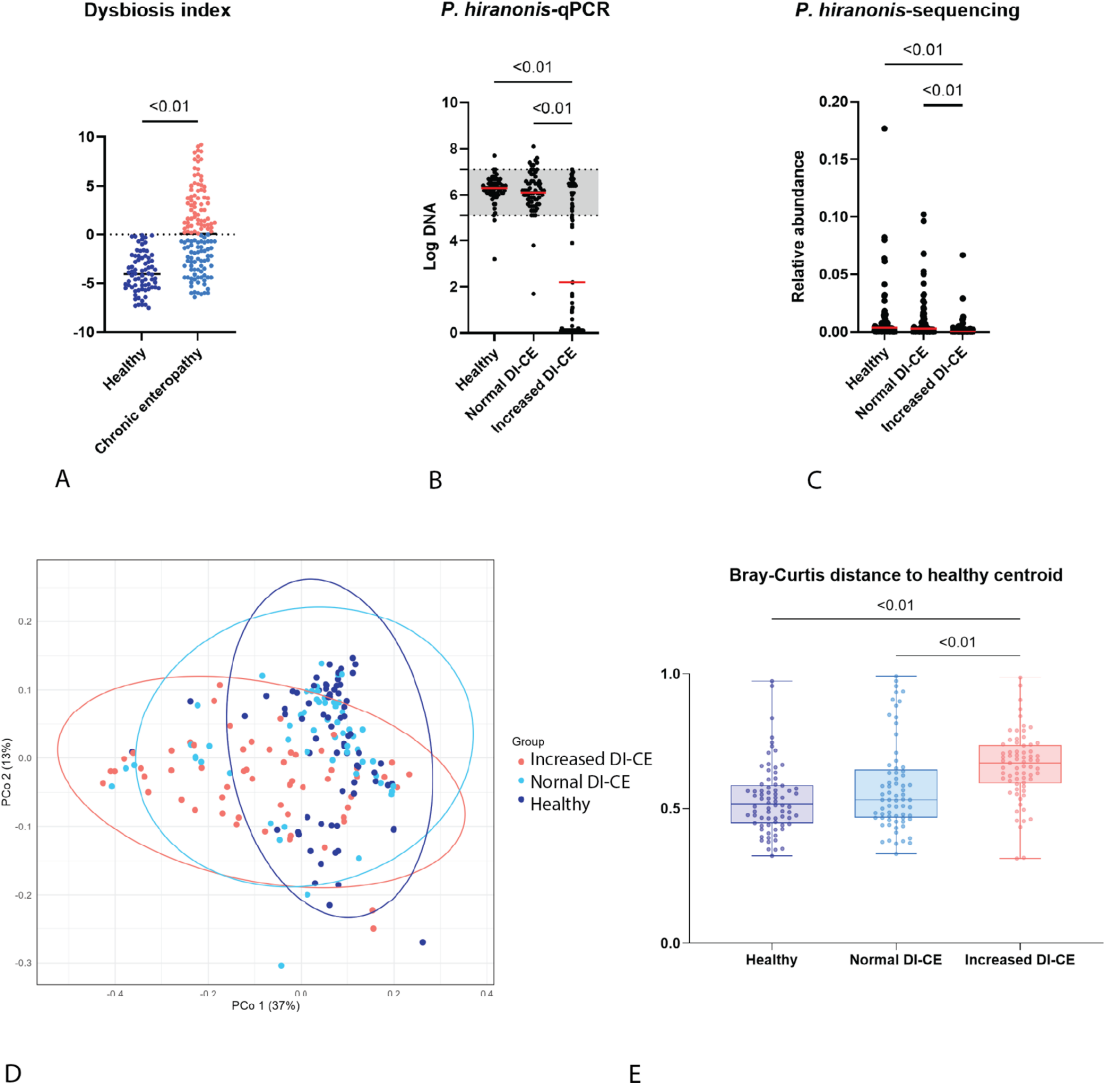
Microbial and functional gene composition. (A) Dogs with CE showed significantly higher dysbiosis index (DI) compared with healthy dogs (*p* < 0.01). The abundances of *Peptacetobacter hiranonis* assessed by qPCR (B) and metagenomic sequencing (C) were significantly lower in the increased DI‐CE group compared with healthy dogs and the normal DI‐CE group (Mann–Whitney *U* test for qPCR, MaAsLin2 for sequencing). However, in a subset of dogs, *P. hiranonis* was detectable by qPCR but undetectable by metagenomic sequencing. (D) PCoA plot of functional gene composition was grouped by clinical status and DI interpretation. Healthy dogs are labeled in dark blue, normal DI‐CE in light blue, and increased DI‐CE in red. The 95% confidence ellipse for each group is color‐coded accordingly. Increased DI‐CE clustered separately from healthy dogs and normal DI‐CE (*F* = 44.0, adjusted‐*p* < 0.01; *F* = 14.9, adjusted‐*p* < 0.01, respectively). A lower magnitude of significance was found between healthy dogs and normal DI‐CE in functional gene composition (*F* = 4.6, adjusted‐*p* = 0.02). (E) Scatter box plot of Bray–Curtis distances to the centroid of the healthy population in microbial gene composition (C). Healthy dogs are labeled in dark blue, normal DI‐CE in light blue, and increased DI‐CE in red. Significant differences were found between increased DI‐CE and the other two groups (adjusted‐*p* < 0.01). Increased DI‐CE: dogs with CE with dysbiosis index above zero; Normal DI‐CE: dogs with CE with dysbiosis index below zero. Only adjusted‐*p* < 0.05 are labeled in the graphs.

The DI overall was significantly higher in dogs with CE (adjusted‐*p* < 0.01), with 50% (57/115) of the dogs with CE having a normal DI (Figure 1A). Both qPCR and sequencing results (Table S7) indicated lower abundances of *P. hiranonis*, alongside higher abundances of *E. coli* and *Streptococcus* in the increased DI‐CE group compared with HC and normal DI‐CE groups. Notably, across qPCR assays, most samples showed detectable bacterial abundances, whereas metagenomic sequencing failed to detect these bacteria in many samples, for example, *P. hiranonis* (Figure 1B,C). Comparisons in individual bacterial taxa between groups are available in Table S3.

A significant difference was found in beta diversity based on the sequencing data in all dogs with CE when compared to HC at the species level (PERMANOVA, Bray–Curtis distance, adjusted‐*p* < 0.01, *F* = 5.4). When categorizing dogs with CE into normal DI‐CE and increased DI‐CE, significant differences were found in all of the comparisons between HC, normal DI‐CE, and increased DI‐CE (adjusted‐*p* < 0.01). However, the differences were more pronounced when comparing increased DI‐CE dogs to HC (*F* = 8.9) or normal DI‐CE groups (*F* = 5.7) compared with the differences observed between normal DI‐CE dogs and HC (*F* = 2.6).

### 3.3 Functional Gene Profile

Statistical significance was found in the beta diversity of functional pathway profiles between HC and CE groups (PERMANOVA, Bray–Curtis distance, adjusted‐*p* < 0.01). When stratifying the CE group by DI, significant differences were found between HC, normal DI‐CE, and increased DI‐CE (adjusted‐*p* < 0.02; Figure 1D). Similar to the taxonomy profiles, the magnitude of differences was larger when comparing increased DI‐CE to HC or normal DI‐CE groups (*F* = 44.0 and *F* = 14.9, respectively) than between normal DI‐CE dogs and HC (*F* = 4.6).

**FIGURE 2:**
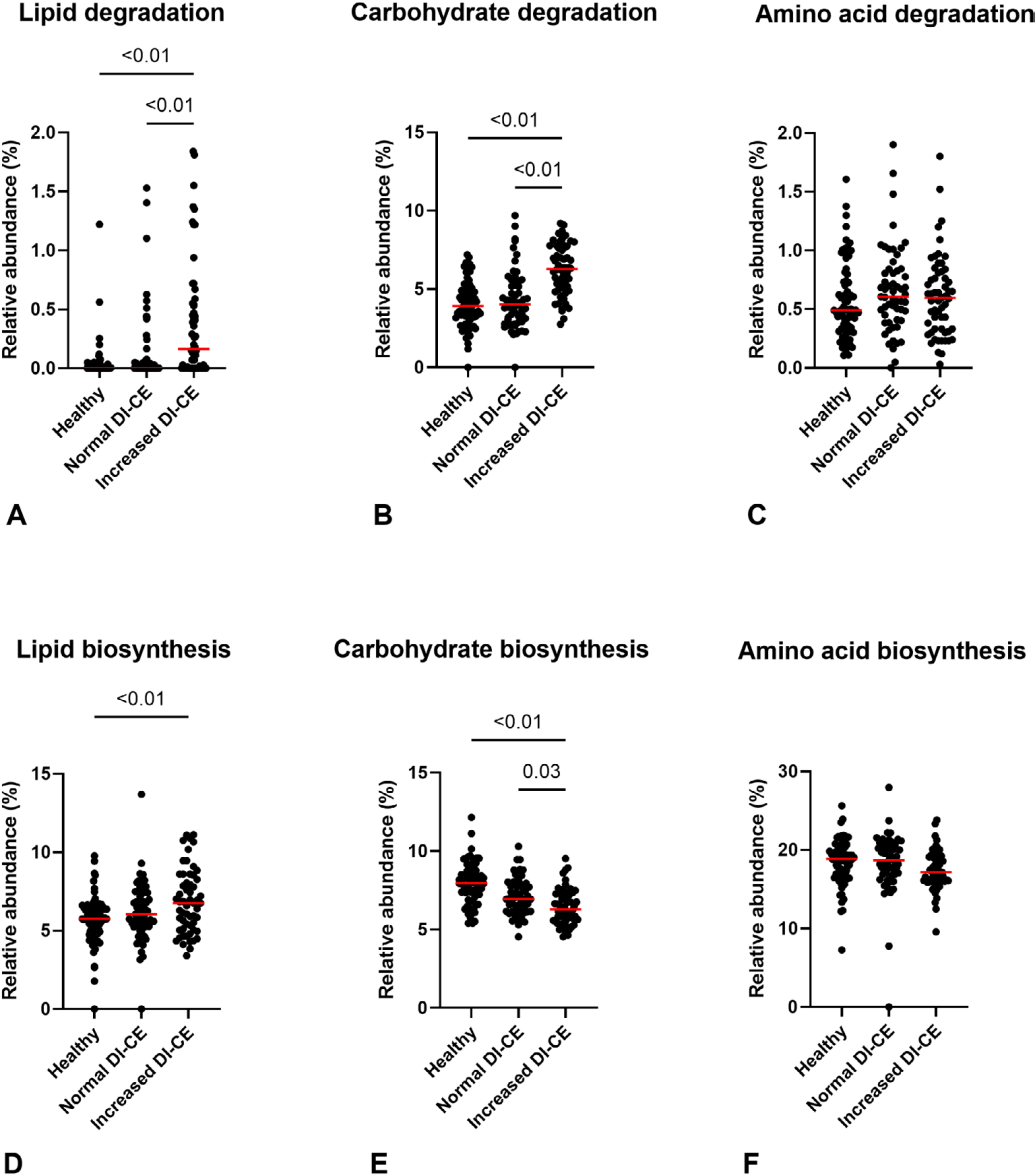
Relative abundances of selected pathways related to macronutrient metabolism. Carbohydrate metabolism (A, D), lipid metabolism (B, E), and amino acid metabolism‐related pathways (C, F). Red lines represent the median of relative abundance. Normal DI‐CE: dogs with CE with dysbiosis index below zero; increased DI‐CE: dogs with CE with dysbiosis index above zero. Only adjusted‐*p* < 0.05 are labeled in the graphs.

To assess higher levels of pathways, we first mapped the pathways back to the parent pathways to which they belonged. Then, we calculated the sum of the relative abundances of pathways for each parent pathway, identifying 39 parent pathways in total. Among all, 10 parent pathways (i.e., aldehyde degradation, aromatic compounds degradation, carbohydrates degradation, fatty acid and lipid degradation, metabolite interconversion, inorganic nutrient metabolism, nucleoside and nucleotide degradation, polyprenyl biosynthesis, protein modification, transport pathways) were enriched and two pathways (i.e., acetate degradation, carbohydrate biosynthesis) were depleted in increased DI‐CE compared with the other groups (adjusted‐*p* < 0.05). Pathways related to metabolism of carbohydrates, lipids, and amino acids are presented in Figure 2. Descriptive statistics of the parent pathways are documented in Table S4.

Next, to analyze the pathways in more detail, we used MaAsLin2 to identify the differential pathways among increased DI‐CE, normal DI‐CE, and HC dogs. Among all 471 pathways, most of the differential abundant pathways were observed in the increased DI‐CE group. Compared with HC, 207 were more abundant and 58 showed less abundance in increased DI‐CE; whereas only 31 were more abundant and 11 showed less abundance in normal DI‐CE. The stratified pathways, showing microbial contributions, then were analyzed. Of 471 pathways, 310 showed at least one bacterial contribution that was significantly higher or lower in increased DI‐CE compared with HC. The main contributors in increased DI‐CE compared with HC or normal DI‐CE aligned with the bacteria that had increased abundance in increased DI‐CE, including *Clostridium perfringens*, *E. coli* , *Streptococcus*, and *Enterococcus*. Compared with increased DI‐CE, in HC and normal DI‐CE groups, *Catenibacterium mitsuokai* , *Holdemanella biformis*, *Bacteroides*, *Blautia*, *Collinsella*, and *Prevotella* were the main contributors. Differential microbial contributions to individual pathways are available in Table S11.

### 3.4 Differential Pathways and Targeted Metabolome

#### 3.4.1 Lipid Metabolism

Seven pathways related to lipid metabolism were enriched in increased DI‐CE. These included four fatty acid oxidation pathways and three long‐chain fatty acid biosynthesis pathways.

**FIGURE 3:**
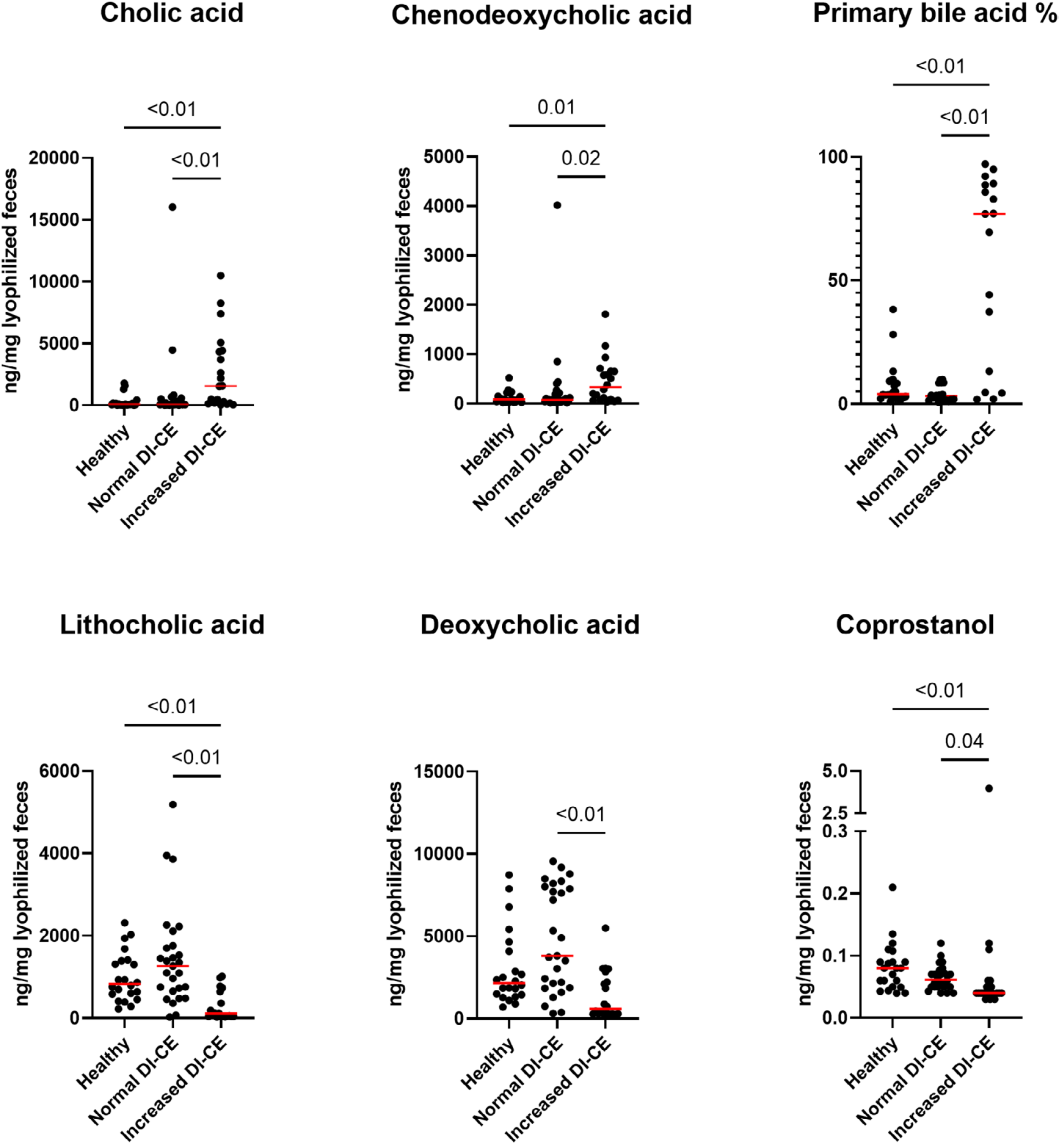
Fecal sterols and bile acids. Fecal concentrations of differentially abundant lipid metabolism‐related compounds in increased DI‐CE compared between HC dogs and normal DI‐CE. Red lines represent the median concentration of each group. Increased DI‐CE: dogs with CE with dysbiosis index above zero; Normal DI‐CE: dogs with CE with dysbiosis index below zero. Only adjusted‐*p* < 0.05 are labeled in the graphs.

In the targeted fecal metabolome analysis, we assessed unconjugated BAs, sterols, and long‐chain fatty acids associated with lipid metabolism in 22 HC dogs and 47 dogs with CE. Among all, the CE group had higher fecal concentrations of cholic acid, palmitic acid, stearic acid, and gondoic acid and lower coprostanol, sitostanol, beta‐sitostanol, and total measured phytosterols compared with HC. Among these, primary unconjugated BAs (i.e., cholic acid and chenodeoxycholic acid) were significantly higher, whereas coprostanol and secondary unconjugated BA (lithocholic acid) were significantly decreased in increased DI‐CE compared with normal DI‐CE and HC dogs (adjusted‐*p* < 0.04; Figure 3). Primary unconjugated BAs were positively correlated with DI (*r* = 0.46), whereas secondary unconjugated BAs (*r* = −0.46) and coprostanol (*r* = −0.34) were negatively correlated with DI (adjusted‐*p* < 0.01). Comparisons in all measured fecal metabolites between groups are available in Table S6. In addition to DI, correlations between individual targeted fecal metabolites and CIBDAI components were assessed (Table S12). Before *p*‐value adjustment, weight loss scores showed moderate positive correlations with 9 of 12 fecal long‐chain fatty acid concentrations.

#### 3.4.2 Carbohydrate Metabolism

Twelve pathways were associated with carbohydrate metabolism and were more abundant in increased DI‐CE. All of these were related to carbohydrate degradation, including pathways for arabinose, sucrose, fucose, and glucarate degradation.

**FIGURE 4:**
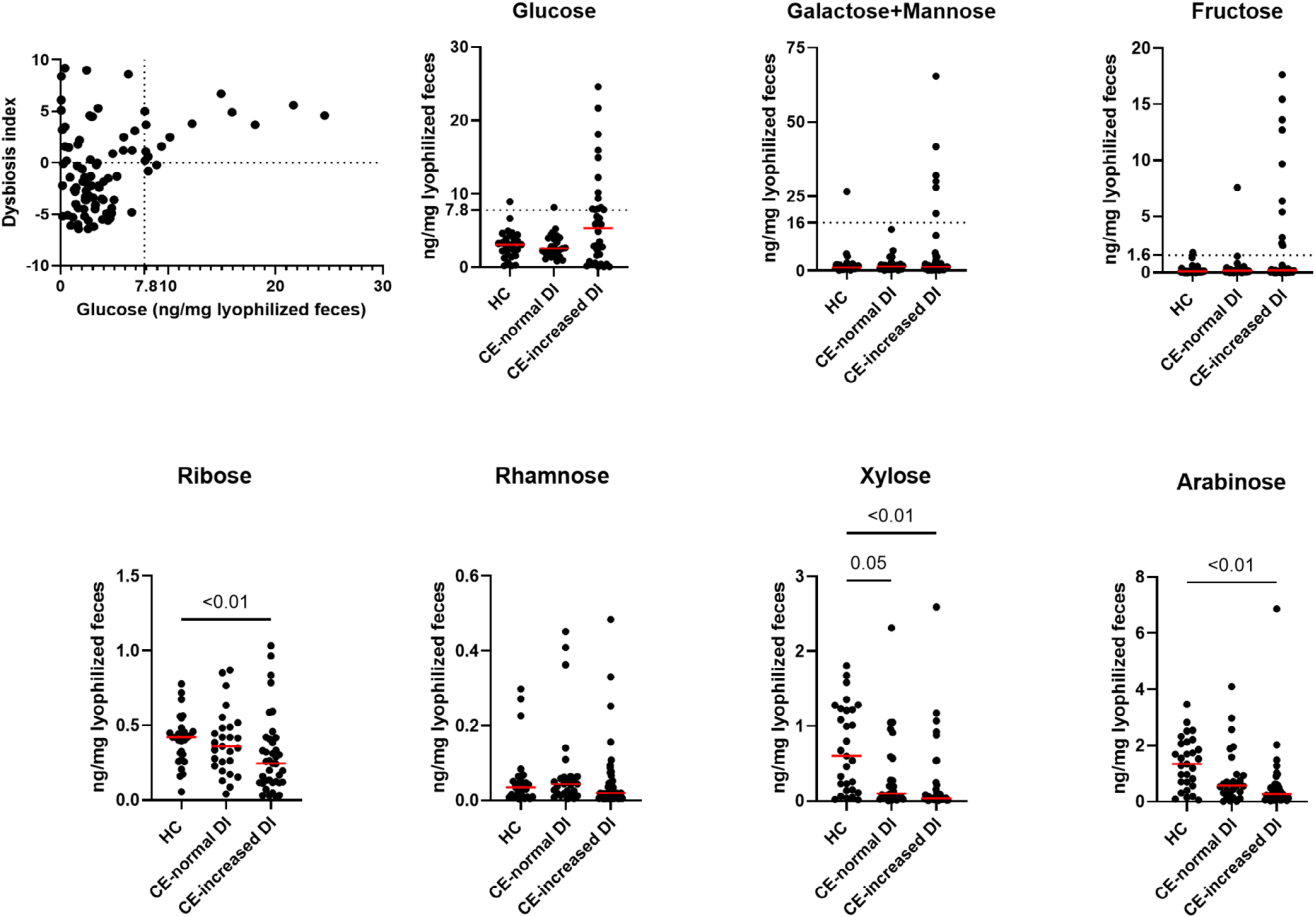
Fecal sugar measurements in dogs. A subset of dogs with CE with increased DI also had fecal glucose, galactose, and mannose, and fructose concentrations above the healthy population. The fecal concentrations of ribose, xylose, and arabinose were significantly lower in dogs with CE with increased DI compared with the healthy group (adjusted‐*p* < 0.01). Red lines represent the median concentration of each group. Increased DI‐CE: dogs with CE with dysbiosis index above zero; Normal DI‐CE: dogs with CE with dysbiosis index below zero. Only adjusted‐*p* < 0.05 were labeled in the graphs.

In the targeted fecal metabolomics, fecal carbohydrate profile was assessed in 29 HC dogs and 63 dogs with CE. Fecal carbohydrates absorbed by simple diffusion (ribose, xylose, and arabinose) were significantly lower in dogs with CE (adjusted‐*p* < 0.03) and negatively correlated with DI (adjusted‐*p* < 0.01). Fecal concentration of transporter‐dependent carbohydrates (glucose, galactose and mannose, and fructose) was increased in a subset of dogs in the increased DI‐CE group but did not reach statistical significance (Figure 4). However, 15/17 (88%) of dogs with a fecal glucose concentration above the 95 percentile of the HC group (7.78 ng/mg lyophilized feces) also had a higher DI (DI > 0; Figure 4).

### 3.5 Targeted Fecal Microbial and Metabolomic Profile

**FIGURE 5:**
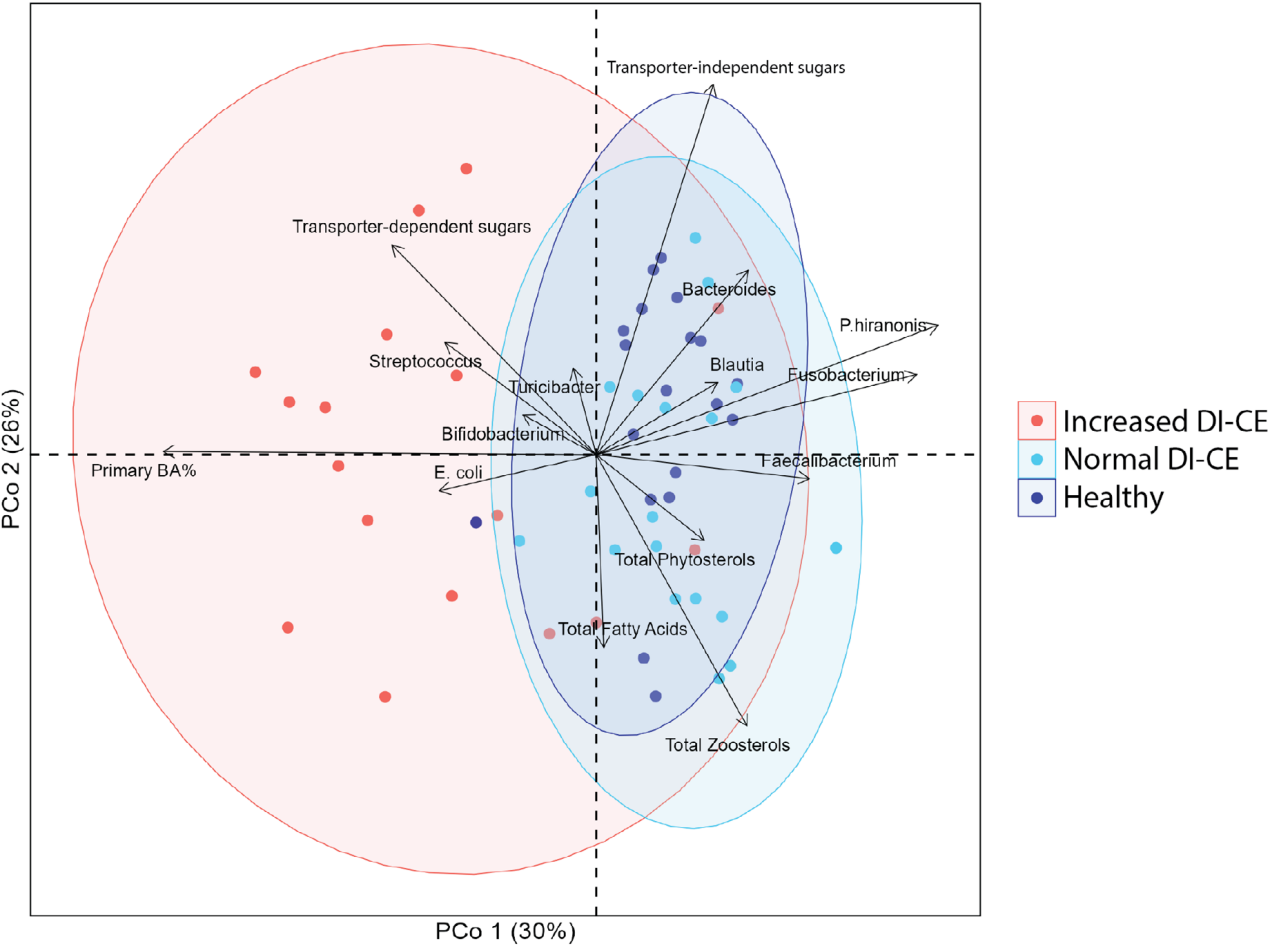
Targeted fecal microbial and metabolomic composition. The PCoA biplot graph shows the targeted fecal microbial and metabolomic compositions grouped by clinical status and DI interpretation. Healthy dogs (dark blue), normal DI‐CE (light blue), and increased DI‐CE (red) are shown with corresponding 95% confidence ellipses. Increased DI‐CE clustered separately from healthy dogs and normal DI‐CE (*F* = 24.4, adjusted‐*p* < 0.01; *F* = 22.9, adjusted‐*p* < 0.01, respectively). Significance was also found between healthy dogs and normal DI‐CE; however, it had a lower variance (*F* = 4.4, adjusted‐*p* < 0.01). All variables were projected as an arrow onto the PCoA plot. The direction of each arrow indicates the gradient of change for that variable, whereas the length represents the strength of its correlation with the ordination axes. Both the direction and length of arrows are derived from the correlation coefficient between each variable and the coordinates. Increased DI‐CE: dogs with CE with dysbiosis index above zero; Normal DI‐CE: dogs with CE with dysbiosis index below zero.

Finally, targeted fecal microbiota and metabolome assessed using qPCR and GC–MS were combined and analyzed (Figure 5). Similar to taxonomy and functional gene profiles from metagenomic sequencing, significances were found in all comparisons among HC, normal DI‐CE, and increased DI‐CE groups (adjusted‐*p* < 0.05). Magnitude of differences was larger when comparing increased DI‐CE to HC or normal DI‐CE (*F* = 24.4 and *F* = 22.9, respectively) than between normal DI‐CE dogs and HC (*F* = 4.4). Variables more abundant in the increased DI‐CE group included primary BA, facultative anaerobes (*Streptococcus* and *E. coli* ), and transporter‐dependent carbohydrates. In contrast, HC and normal DI‐CE groups had larger amounts of secondary BA and strict anaerobes (i.e., *Turicibacter*, *Bacteroides*, *Faecalibacterium*, *Blautia*, *Fusobacterium*, and *P. hiranonis*). Differences between HC and normal DI‐CE groups were associated with the fecal concentration of total fatty acids and zoosterols. The statistical analysis results regarding each feature and the coordinates are available in Table S10.

## 4 Discussion

Chronic enteropathy in dogs is a multifactorial disease with dysregulated immune response, genetic predisposition, and intestinal microbiome playing roles [39]. However, major changes in fecal microbial and metabolomic profiles are not observed in every dog with CE [2, 4, 8, 18, 19, 20, 21]. Additionally, the microbial functional gene profiles have not been well explored. Understanding the microbiota's potential capabilities, rather than solely its community structure, can be essential for recognizing the host–microbial interaction and its role in disease in dogs with CE [40]. Addressing this knowledge gap and building upon existing understanding, our study described the fecal microbiota compositions, fecal microbial gene profiles, the targeted fecal metabolome, and their correlation with fecal microbiota.

In previous studies, altered microbial taxonomy has been reported in feces [4, 8, 29, 41] and intestinal specimens (e.g., duodenal mucosa [2, 42, 43], ileal mucosa [44], colonic mucosa [44, 45]) in dogs with CE using either untargeted sequencing or targeted assays. However, these studies also found considerable overlap between healthy and CE dogs. In dogs with CE, the current clinical classification is based on clinical response to treatments, yet at present, no specific biomarkers, clinical signs, or scoring systems have been established to distinguish among the clinical phenotypes of CE in dogs, and likely significant overlap in these treatment categories occurs [46]. Similarly, in our data, no significant differences were found when categorizing dogs according to their treatment response (Table S5). This lack of differentiation in microbiome composition and function aligns with previous research, which reported no significant differences in global richness, diversity, and microbial composition between dogs with food‐responsive enteropathy and those with IBD [47].

Several studies suggested that the DI, a targeted assay for assessing the fecal microbiota in dogs, can predict the overall shifts in the microbiome observed on shotgun metagenomic sequencing [17, 27, 28]. In agreement, we found that normal DI‐CE shared similar taxonomic profiles with HC dogs, whereas increased DI‐CE had distinct microbial profiles compared with normal DI‐CE and HC. We also found similar patterns in the beta diversity of microbial functional gene compositions. Normal DI‐CE exhibited similar functional gene profiles to HC, whereas increased DI‐CE had a more shifted functional gene composition. Additionally, we stratified dogs with both microbial functional gene and targeted metabolomics data into five groups based on clinical status and DI values: HC, and CE dogs with DI < 0, 0–2, 2–5, and > 5. We observed increasing *R* ^2^ and *F* values from PERMANOVA analyses, indicating that higher DI scores were associated with more divergence from the HC group (Table S9). When examining the pathways both at a lower and higher magnification, most of the differentially abundant pathways were found in increased DI‐CE. All results suggest that increased DI‐CE had a different gut microbial composition and microbial functional potentiality compared with normal DI‐CE and HC dogs. The variance in the degree of microbiome shifts may suggest different pathophysiology in dogs with CE, specifically between those animals with fecal microbiome compositions similar to HC and those presenting with distinct compositions at taxonomic and functional levels. Although DI was correlated with the overall shifts in fecal microbiome composition assessed by DNA shotgun sequencing and targeted fecal metabolomic profiles, it may not capture more subtle changes.

It remains unclear whether the differences in the microbial taxonomic and functional gene profiles were the causes of the disease or the consequences of the abnormal intestinal environment (e.g., maldigested or malabsorbed nutrients, increased inflammatory cytokines, oxidative stress). We found that, compared with other groups, a subset of increased DI‐CE presented with more carbohydrate degradation‐related pathways and fecal concentrations of transporter‐dependent carbohydrates and fewer carbohydrate biosynthesis‐related pathways. We hypothesized that excessive glucose, galactose, mannose, and fructose in the gut lumen resulted from the altered gut environment, leading to adaptive changes in the bacterial community or their metabolic activity [48, 49]. Possible explanations for carbohydrate‐induced gastrointestinal signs in humans include ingesting carbohydrates beyond physiological limits and decreased absorption because of transporter defects or mucosal damage [50, 51, 52]. Detailed dietary histories were unavailable for most dogs, but all dogs were fed commercially available diets and we assume that the majority of these diets were complete and balanced and had an Association of American Feed Control Officials (AAFCO) adequacy statement for adult maintenance. Consequently, the first hypothesis is less likely to apply to our CE population, leaving us to speculate on the involvement of the latter in the pathophysiology of CE in dogs.

Furthermore, in humans, clinical improvement has been observed in some patients with irritable bowel syndrome (IBS) after adopting diets low in fermentable oligosaccharides, disaccharides, monosaccharides, and polyols (FODMAP), which are low in fermentable carbohydrates. Although the effects of a low FODMAP diet in dogs with CE have not yet been studied, it has been reported that the sources and types of dietary carbohydrates can influence fecal quality [53]. Therefore, future studies should investigate the effect of modifying the amount or sources of dietary carbohydrates in increased DI‐CE, especially those with altered fecal carbohydrate profiles.

Despite no significant differences found when summing up amino acid biosynthesis‐related pathways, most fecal amino acids were shown to increase in humans with IBD [54, 55], and dogs with ce [ 29]. However, in one study using a targeted high‐performance liquid chromatography (HPLC) approach measuring fecal amino acids, except for increased fecal tryptophan concentration in a subset of dogs with CE, no other significant findings were reported [56]. In our study, the pathway of l‐tryptophan biosynthesis (PWY6629) was over‐represented in increased DI‐CE compared with the other groups, aligning with previous studies showing increased fecal tryptophan concentration in a subset of dogs with ce [ 29, 56] and humans with IBD [54]. The authors hypothesized that the increased fecal amino acid concentration might be caused by the following mechanisms: In our study, some individual amino acid biosynthesis pathways were increased in increased DI‐CE, indicating increased bacterial production. For amino acids where bacteria biosynthesis pathways were underpresented, intestinal malabsorption caused by downregulation of intestinal amino acid transporters [57], protein loss through colonic leakage [54, 55, 58], and increased catabolism of protein by the host [54, 55], as suggested by literature in human medicine, were considered as potential explanations.

Alterations of fecal BA profiles have been associated with CE in dogs [18, 19, 20]. *P. hiranonis* has been identified as the main BA converter using the 7α‐dehydroxylation pathway [59]. Aligned with prior studies [18, 20], fecal concentrations of unconjugated secondary BAs, lithocholic acid and deoxycholic acid, were decreased in a subset of dogs with CE, in our study, specifically in increased DI‐CE. Also, fecal abundance of *P. hiranonis* was significantly lower in increased DI‐CE. However, we did not identify any BA‐related genes or pathways in our sequencing data, possibly because of insufficient sequencing depth to identify rare genomes.

Consistent with several published reports, *E. coli* and *Streptococcus* were enriched in a subset of dogs with CE [21, 29], and the relative abundances of these bacteria also were increased in increased DI‐CE in our study. *E. coli* and *Streptococcus* are heterotrophic bacteria, feeding either on starch and sugars (saccharolytic) or proteins and peptides (proteolytic). On the other hand, bacteria predominant in HC and normal DI‐CE (e.g., *P. copri* [ 60, 61] and *C. mitsuokai* [ 62, 63]) have been characterized as saccharolytic but not proteolytic. The enrichment of *E. coli* and *Streptococcus* and depletion in *P. copri* and *C. mitsuokai* in increased DI‐CE further support our speculation that intestinal amino acid malabsorption might have a role in CE. An increased amino acid concentration in the intestinal lumen can cause both dysbiosis and gastrointestinal signs.

Our study had some limitations. We analyzed only single time points [64]. Secondly, our study included samples from university teaching hospitals and referral hospitals. Therefore, it is possible our population targeted those patients at a more advanced disease stage or needing multimodal treatments resulting in 29% of food‐responsive enteropathy in our population. Additionally, samples were collected over 8 years and stored at −20°C or −80°C before analysis. A more diverse type of fecal BAs in dogs and cats has been presented in two recent studies [65, 66]. Although the focus of this study was to investigate the major functionality, one being the conversion of unconjugated primary and secondary BAs, a more comprehensive fecal BA profile would extend our knowledge of the dogs with CE with different microbial profiles. Diets, modulating the protein or fiber content, have been suggested to substantially impact clinical signs, fecal taxonomy composition, microbial functional gene profiles, and metabolome in dogs [13, 67, 68, 69, 70, 71] and cats [72, 73, 74, 75]. Unfortunately, in our study, detailed dietary history was not collected upon inclusion. However, two studies, using 16S rRNA gene sequencing and DI, respectively, have shown that clinically healthy dogs only exhibited minimal fecal microbiota changes in response to dietary interventions compared with those caused by gastrointestinal diseases [76, 77]. The extent of microbiome shifts induced by diet is considered minor. For instance, one study using 16S rRNA gene sequencing with unweighted analysis found no significant changes before and after diet interventions in both the food‐responsive enteropathy group and healthy dogs, but observed significant differences between diseased and healthy groups [8]. Similarly, although age has been shown to affect fecal microbial and metabolomic profiles, the effect is relatively minor. One study reported no significant differences in fecal microbial beta diversity and DI among dogs of different age groups [11], and another found only minor fecal microbial changes [78]. Consistent with these findings, no significant differences between age groups were found in the functional gene profile and DI in our study (Table S8). Additionally, certain genes, such as those related to BA metabolism, were not detected in our dataset. This observation suggests that a deeper sequencing depth might be required for more comprehensive detection and analysis. However, the cost of deep sequencing in a sample size similar to that used in our study may be prohibitive. In addition, a previous study reported no distinct separation between shallow and deep sequencing when performing beta diversity clustering [79]. Furthermore, in our study, metagenomic sequencing failed to detect certain bacterial species in some samples identified by qPCR assays. This discrepancy suggests that, as an untargeted approach, metagenomic sequencing is suited for discovery purposes. However, qPCR may be a more reliable method in quantification and diagnostic applications. Finally, microbial functional gene profiles measured by metagenomics sequencing represent the potential functionality of the inhabitants. Actual gene expression is needed for future study.

Notwithstanding these limitations, our study suggests that in dogs with CE, those with severe microbial shifts also have altered host and microbial metabolic functions. Thus, we suspect different pathophysiology behind increased DI‐CE and normal DI‐CE. Future studies regarding the mechanism and the corresponding treatment of both groups of dogs with CE are needed. Absorption and digestion studies utilizing nutrient isotopes or investigating the expression of intestinal nutrient receptors are necessary next steps to understand the mechanisms behind the alteration of fecal metabolome profiles in dogs with CE.

## Disclosure

Authors declare no off‐label use of antimicrobials.

## Ethics Statement

Authors declare no institutional animal care and use committee or other approval was needed. Authors declare human ethics approval was not needed.

## Conflicts of Interest

The authors Chih‐Chun Chen, Rachel Pilla, Linda Toresson, Chi‐Hsuan Sung, Amanda B. Blake, Bruna Correa Lopes, Jonathan Turck, Patricia Eri Ishii, Paula R. Giaretta, M. Katherine Tolbert, and Jan S. Suchodolski are affiliated with the Gastrointestinal Laboratory, Texas A&M University, which offers gastrointestinal assays on a fee‐for‐service basis. Albert E. Jergens has served as a consultant and speaker for Purina, Hill's, Pharma LLC, and VETmAB BIO.

## Supporting information


**Table S1:** Study population.
**Table S2:** Inter and intra‐assay variability of fecal carbohydrates concentration.
**Table S3:** Comparison of dysbiosis index and individual taxa assessed by qPCR.
**Table S4:** Statistic results of shotgun sequencing Level 3 pathways.
**Table S5:** Dysbiosis index and functional gene profile comparisons between different phenotypes of CE.
**Table S6:** Targeted fecal metabolomics.
**Table S7:** Differential bacteria from metaenomic sequencing at species and genus level.
**Table S8:** Dysbiosis index and functional gene profile comparisons between different ages.

**Supporting Information S9:** Contribution of species to different metabolic pathways.
