# Supplementary material for: Microbial Gene Profiling and Targeted Metabolomics in Fecal Samples of Dogs With Chronic Enteropathy With or Without Increased Dysbiosis Index
Source: J Vet Intern Med. 2025 Aug 14;39(5):e70199. doi: 10.1111/jvim.70199 (PMC12351495; doi:10.1111/jvim.70199)

Supplementary S9. Contribution of species to different metabolic pathways

Glucose and glucose-1-phosphate degradation (A) and glycolysis III (from glucose) (B) were 2 of the pathways identified in this dataset associated with glucose metabolism. The former pathway was over-presented in increased DI-CE compared to the other two groups (adjusted-*P* < .01); the latter pathway showed no difference in its relative abundance among groups (adjusted-*P* > .05). *Escherichia coli* was the most important contributor to the glucose and glucose-1-phosphate degradation pathway. *Prevotella copri* and *Catenibacterium mitsuokai* were the main contributors to the glycolysis III pathway in normal DI-CE and HC, while *Streptococcus lutetiensis* and *E. coli* were the main contributors to increased DI-CE.


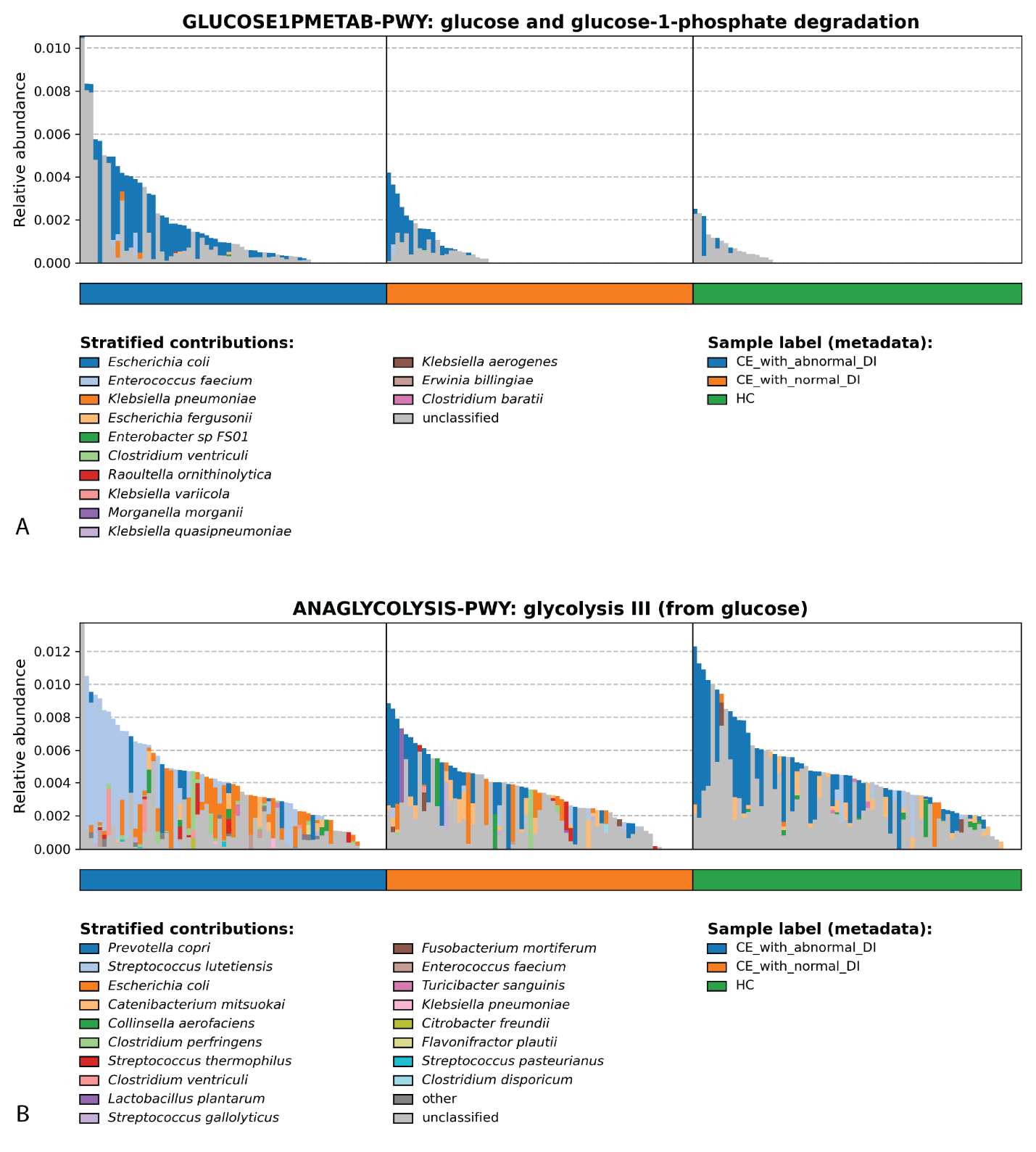

Supplement: Supplementary file 2 — Supporting Information S9: Contribution of species to different metabolic pathways. [file JVIM-39-e70199-s002.docx]
